# Where there is no morphine: The challenge and hope of palliative care delivery in Tanzania

**DOI:** 10.4102/phcfm.v6i1.549

**Published:** 2014-11-14

**Authors:** Kristopher Hartwig, Mervyn Dean, Kari Hartwig, Paul Z. Mmbando, Abduraoof Sayed, Elma de Vries

**Affiliations:** 1 Evangelical Lutheran Church in Tanzania, Tanzania; 2 Consulting Palliative Care Physician, Tanzania; 3 College of Health Sciences, Walden University, United States; 4 Department of Community Health, University of Cape Town, South Africa; 5 Division of Family Medicine, University of Cape Town, South Africa

## Abstract

**Background:**

In Tanzania, a country of 42 million, access to oral morphine is rare.

**Aim:**

To demonstrate the effectiveness of palliative care teams in reducing patients’ pain and in increasing other positive life qualities in the absence of morphine; and to document the psychological burden experienced by their clinical providers, trained in morphine delivery, as they observed their patients suffering and in extreme pain.

**Setting:**

One hundred and forty-five cancer patients were included from 13 rural hospitals spread across Tanzania.

**Method:**

A mixed method study beginning with a retrospective quantitative analysis of cancer patients who were administered the APCA African POS tool four times. Bivariate analyses of the scores at time one and four were compared across the domains. The qualitative arm included an analysis of interviews with six nurses, each with more than five years’ palliative care experience and no access to strong opioids.

**Results:**

Patients and their family caregivers identified statistically significant (p < 0.001) improvements in all of the domains. Thematic analysis of nurse interviews described the patient and family benefits from palliative care but also their great distress when ‘bad cases’ arose who would likely benefit only from oral morphine.

**Conclusion:**

People living with chronic cancer-related pain who receive palliative care experience profound physical, spiritual and emotional benefits even without oral morphine. These results demonstrate the need for continued advocacy to increase the availability of oral morphine in these settings in addition to palliative care services.

## Abstrait

**Là où il n'y a pas de morphine: les défis et espoirs de la fourniture de soins palliatifs en Tanzanie**.

**Contexte:**

En Tanzanie, un pays de 42 millions d'habitants, l'accès à la morphine par voie orale est rare.

**But:**

Montrer l'efficacité des équipes de soins palliatifs dans la réduction de la douleur des patients et l'amélioration d'autres qualités de vie en l'absence de morphine; et documenter la charge psychologique vécue par les prestataires cliniques formés à l'utilisation de la morphine lorsqu'ils observaient chez leurs patients une souffrance et une douleur extrême.

**Cadre:**

Cent quarante-cinq patients atteints du cancer venant de 13 hôpitaux ruraux répartis dans toute la Tanzanie ont été étudiés.

**Méthode:**

Une étude à méthode mixte commençant par une analyse quantitative rétrospective des patients atteints du cancer à qui l'outil Afrique POS APCA avait été administré à quatre reprises. Des analyses bivariées des résultats au moment un et quatre ont été comparées pour tous les domaines. Le volet qualitatif comprenait une analyse d'entretiens avec six infirmiers/-ières ayant chacun plus de cinq ans d'expérience en soins palliatifs et sans accès à des opioïdes puissants.

**Résultats:**

Les patients et leurs soignants familiaux ont identifié des améliorations statistiquement positives (*p* < 0,001) dans tous les domaines. L'analyse thématique des entretiens auprès des infirmiers/-ières a décrit les bienfaits des soins palliatifs pour le patient et sa famille, mais aussi leur grande détresse lorsque des « mauvais cas » surviennent pour qui seule la morphine par voie orale pourrait probablement apporter un soulagement.

**Conclusion:**

Les personnes vivant avec une douleur chronique liée au cancer qui reçoivent des soins palliatifs sont physiquement, spirituellement et émotionnellement profondément soulagées, même sans morphine par voie orale. Ces résultats montrent le besoin de plaidoyer permanent en faveur de l'accroissement de la disponibilité de morphine par voie orale dans ces contextes en plus des services de soins palliatifs.

## Introduction

In rural Tanzania, as in much of the developing world, diagnosis of cancer occurs late in the clinical course of disease, with the result that palliative care is often the best and only option for care.^1, 2^ Yet, in low- and middle-income countries, few palliative care services are available as a result of a shortage of trained professionals, a lack of opioids for pain treatment and limited integration into standard medical care.^3, 4^

The World Health Organization (WHO) defines palliative care as being a holistic approach for patients and their families who are living with life-threatening diseases, particularly emphasising the treatment of pain.^5^ Oral morphine is widely accepted as being the first-line drug for treatment of moderate- to severe cancer pain,^6^ but it is of limited availability in much of the world despite ongoing advocacy for recognition of pain relief as a basic human right.^7, 8^ The result is that many people living with cancer are left to die at home, or in hospital, with no effective way to relieve their physical suffering. Efforts to provide palliative care without access to oral morphine have lacked credibility. Anne Merriman, a pioneer in African palliative care, observed that ‘palliative care without morphine is only supportive care… and lack of access to morphine is torture.’^9^

In 2010, the WHO estimated that over 14 000 of Tanzania's population of over 40 million will die of cancer each year.^10^ Due to the vast distances involved, the immobility of the very sick and limited training of clinical staff, very few patients will have access to a palliative care service.

Tanzania's Pharmaceutical Board requires individual hospitals to apply for a licence to administer morphine; few hospitals currently have this licence and even when such a service is available the supply of oral morphine may be limited as a result of stringent reporting and access regulations. Overall consumption of morphine at the national level remains very low,^11^ presumably because of low availability (in 2010, only four hospitals had licences to dispense morphine) and limited prescription, even when available.

Palliative care is relatively new to Tanzania. Initial efforts began in the early 1990s at the Ocean Road Cancer Institute in Dar es Salaam, but it was not until this century that other organisations began concerted efforts with regard to provision of palliative care. By 2002 there were three further sites: PASADA (Pastoral Activities and Services for people with AIDS, Dar es Salaam Archdiocese), Muheza Hospice (Tanga Region) and Selian Hospice (Arusha Region).^12^ What is the experience of the few palliative care patients and their providers in settings with no morphine? There is little available in the literature to document the value and limitations of palliative care in these settings. Through a retrospective study of data gathered from 2009 to 2010, we aim to address this gap.

## Background to the study

**FIGURE 1:**
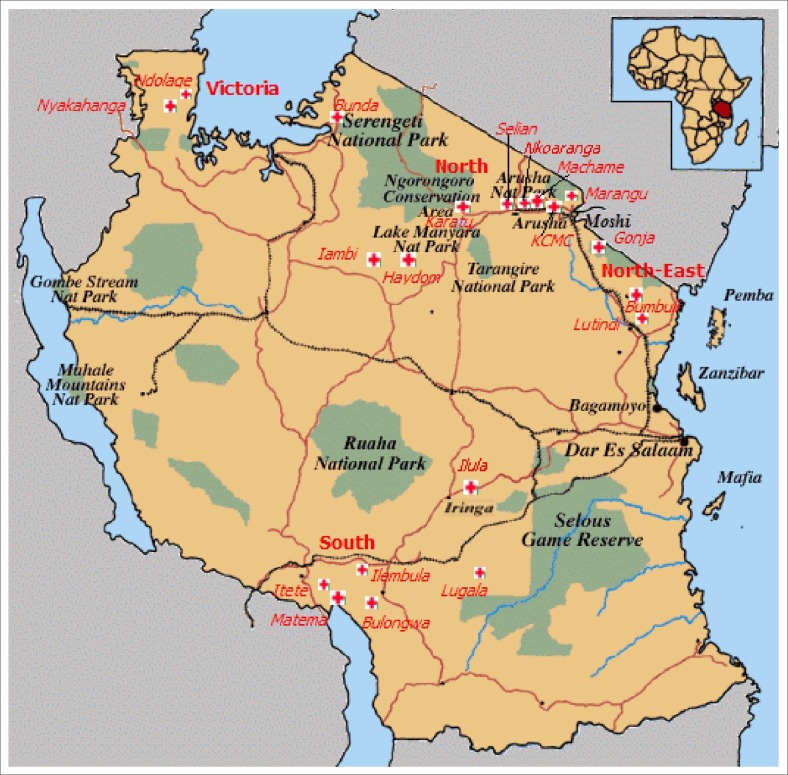
Location of Evangelical Lutheran Church in Tanzania hospitals on map of Tanzania. *Note*: Each cross represents an Evangelical Lutheran Church in Tanzania hospital. Four additional hospitals have opened or are in the process of opening, three located in the northeast and one in the far northwest.

In 2004, the Evangelical Lutheran Church in Tanzania (ELCT) began a programme in palliative care, emulating the growth of Selian's service. Using a hospital-based approach, trained palliative care teams were developed through 18 rural ELCT hospitals, scattered widely throughout the country (Figure 1). In 2007, the ELCT programme, in collaboration with the Foundation for Hospices in Sub-Saharan Africa (FHSSA) and the African Palliative Care Association (APCA), received funding from the US government's President's Emergency Program for AIDS Relief (PEPFAR) in order to initiate the CHAT program (Continuum of care for people living with HIV/AIDS in Tanzania)^13, 14, 15, 16^ in 13 of the ELCT hospitals, which varied in size from 60 to 400 beds and served catchment areas of between 100 000 and 400 000 people.

CHAT ran from 2007 to 2010 and comprised a supervising palliative care team and mobile hospital-based teams with vehicle transport that provided home-based care (HBC) and monitored pain and the quality of care systematically with a validated assessment tool, the APCA African Palliative Outcome Scale (POS). CHAT funders allowed all patients with terminal illness, including cancer, admission to the programme.

The palliative care teams had been trained to treat moderate to severe pain using the WHO Analgesic Ladder^17^ within the limits of the drugs they had at their disposal. At the time of the study oral morphine was not accessible in most of rural Tanzania, including the 13 sites that were part of CHAT. As a result, the practice of pain management faced significant challenges requiring each palliative care team clinician and nurse to become proficient in the systematic use of the available analgesics, of which tramadol was the most potent.

Studies have very clearly shown the benefit of HBC programmes for people living with HIV,^18^ but there is little information about such care for cancer patients where oral morphine is lacking. While it might be intuitive that the general well-being of any patient would be enhanced by attention from a palliative care team, is the team's efficacy compromised by the absence of morphine? Although tramadol has been studied in the treatment of cancer pain, it has been in the context of more potent options.^19, 20, 21, 22^ The teams administered tramadol to a maximum dose of 400 mg/d to all patients with moderate to severe pain and combined it according to patient response and tolerance with ibuprofen, paracetamol and/or amitriptyline.

The aim of this paper is twofold. First is to document, using the POS indicators, the value of palliative care for patients living with cancer but without access to morphine. Second is to document the psychological burden experienced by their clinical providers, who are trained in morphine delivery but have no access to the drug, as they observe their patients suffer in pain.

## Research methods and design

The study was conducted from January 2009 to December 2010, employing a combination of quantitative and qualitative methods. The quantitative component comprised a retrospective cohort analysis of 145 cancer patients across the 13 hospital sites that did not have access to oral morphine. The qualitative arm involved face-to-face interviews with nurses from the hospital sites.

### Retrospective cohort analysis

The POS, designed to be administered four times over two to four weeks, has been validated in several of the African languages for people over the age of 18 years and for a variety of disease conditions.^23, 24^ For our study a Swahili version of the POS – Swahili is the official language of Tanzania – was used by the attending nurse with translation into the local dialect or language where necessary. The POS contains seven questions for the patient and three questions for family caregivers. Patients identify physical symptoms, including pain and psychological status, on a scale of zero to five. (See Table 1 for the English version of the APCA African POS).

**TABLE 1:** African Palliative Care Association Palliative Outcome Scale (APCA POS).

Questions	Rating
**Ask the patient**	
Q1. Please rate your pain (from ) = no pain to 5 = worst/overwhelming pain) during the last 3 days	0 (no pain)– 5 (worst/overwhelming pain)
Q2. Have any other symptoms (e.g. nausea, coughing or constipation) been affecting how you feel in the last 3 days?	– 5 (overwhelmingly)
Q3. Have you been feeling worried about your illness in the past 3 days?	0 (not at all)– 5 (overwhelming worry)
Q4. Over the past 3 days, have you been able to share how you are feeling with your family or friends?	0 (not at all)– 5 (yes, I've talked freely)
Q5. Over the past 3 days, have you felt that life was worthwhile?	0 (no, not at all)– 5 (yes, all the time)
Q6. Over the past 3 days, have you felt at peace?	0 (no, not at all)– 5 (yes, all the time)
Q7. Have you had enough help and advice for your family to plan for the future?	0 (not at all)– 5 (as much as wanted)
**Ask the family carer**	
Q8. How much information have you and your family been given?	0 (none)– 5 (as much as wanted)N/A
Q9. How confident does the family feel caring for ___________________?	0 (not at all)– 5 (very confident)N/A
Q10. Has the family been feeling worried about the Client over the last 3 days?	0 (not at all)– 5 (severe worry)N/A

Inclusion criteria for the study were people over 18 years of age living with cancer for whom three or four POS assessments had been performed between January 2009 and December 2010. The data were taken from the reports which were submitted monthly to the central ELCT office as part of the routine data collected by the CHAT project. Each hospital assigned patient numbers and all POS files used this numbering system to protect patient confidentiality. All records were kept in a locked file with patient identifiers in a separate file.

The APCA POS data were entered into Microsoft Excel and analysed using Stata version 11 (StataCorp. 2009. Stata Statistical Software Release 11. College Station, TX: StataCorp LP). Prior to data analysis, low-scoring positive scores, such as a ‘2’ for minimal worry were reversed numerically so that all negative scores were skewed toward the higher numbers and could be compared with ease. To assess statistical significance over time, scores from the first and last visits were compared for each question. The Shapiro-Wilk test indicated that the key outcome variables, pain and other domains as measured by the POS were not distributed normally, thus the non-parametric Wilcoxon-signed rank test was applied. The statistical level of significance was set at *p* < 0.05.

### Qualitative analysis

In order to assess clinical providers’ perspectives on quality of pain management for cancer patients in settings without morphine, the first author conducted face-to-face interviews with six nurses from six of the CHAT hospitals. We selected nurses using criterion sampling,^25^ specifically, years of experience and palliative care training. Three of the nurses held diplomas in palliative care whilst the other three each had at least seven years of experience working in palliative care. All six nurses had received training from APCA, Nairobi Hospice or Hospice Uganda, each being a well-established programme in East Africa.

We used a semi-structured interview guide to conduct each interview and tape-recorded all sessions. The core questions asked the nurse to (1) describe in detail the case of a patient with cancer who, whilst under their team's care, had poor pain control; (2) describe the case of a patient with cancer with very good pain control; and (3) reflect on the value of palliative care in the setting of no access to oral morphine for chronic pain control.

The first author conducted all the interviews; five in Swahili and one in English. The same author transcribed all of the interviews and translated the Swahili interviews into English prior to data analysis. Two authors (K.H. and M.D.) conducted a thematic analysis of the interview transcripts with particular attention to the nurses’ perspectives on their patients’ pain control and quality of life and their own experience in coping as professionals with no access to morphine. The two analysts used an iterative process of independently identifying themes and quotes within the interviews and then comparing results. The final set of themes emerged after discussion and further iterative analysis of the transcripts.

## Ethical considerations

The University of Cape Town (UCT) and the National Institute of Medical Research (NIMR) of Tanzania gave ethical approval for the study (UCT: HREC REF 319/210, IRB Number IRB00001938; NIMR: NIMR/HQ/R.8a/Vol. 1X/1025). Each nurse provided written voluntary consent to be interviewed.

## Results

Of the 153 satisfactorily-completed POS records, eight patients had died before the third visit, leaving 145 records for inclusion in the study. Women made up 87 (60%) of the patients and had an average age of 57 compared with men at 56. Given the limited diagnostic procedures and treatments available in rural areas precise diagnoses, including co-morbidities, are rarely available so we did not attempt to collect such data.

The results for each domain of the POS showed a statistically-significant change from first to last visit (*p* < 0.001). The worst problem at each visit was pain, with the average first visit pain score being 3.8 (slightly higher for men), which compared with the next worst score of 3.4 for patient anxiety. By the last visit the average pain score for both men and women was 2.3, similar to that of several other symptoms (Table 2). Table 3 illustrates improvement over time in every domain for both patients and family members.

**TABLE 2:** Gender comparisons

Gender	Number	Average Age	Visit 1 Average Pain	Visit 4 Average Pain	*p-*value
Female	87 (60%)	57	3.71	2.31	< 0.001
Male	58 (40%)	56	4.02	2.31	< 0.001
Total	145	–	–	–	–
Average	–	57	3.83	2.31	< 0.001

**TABLE 3:** Summary statistics of Palliative Outcome Scale scores at each visit (1 to 4).

Questions	Visit	*N*	Mean	SD	Median	Min	Max	*p*-value
Q1. Please rate your pain (from 0 = no pain to 5 = worst/overwhelming pain) during the last 3 days	1	145	3.8	1.0	4	1	5	< 0.001
	2	145	3.1	1.0	3	1	5	
	3	145	2.7	1.0	3	0	5	
	4	145	2.3	1.2	2	0	5	
Q2. Have any other symptoms (e.g. nausea, coughing or constipation) been affecting how you feel in the last 3 days?	1	145	2.6	1.5	3	0	5	< 0.001
	2	145	2.0	1.4	2	0	5	
	3	145	1.7	1.3	2	0	5	
	4	144	1.5	1.3	2	0	5	
Q3. Have you been feeling worried about your illness in the past 3 days?	1	145	3.4	1.6	4	0	5	< 0.001
	2	144	2.7	1.4	3	0	5	
	3	144	2.3	1.3	2	0	5	
	4	143	2.1	1.4	2	0	5	
Q4. Over the past 3 days, have you been able to share how you are feeling with your family or friends?	1	145	3.5	1.7	4	0	5	< 0.001
	2	144	3.8	1.3	4	0	5	
	3	145	4.1	1.1	4	1	5	
	4	145	4.2	1.1	5	1	5	
Q5. Over the past 3 days have you felt that life was worthwhile?	1	145	2.0	1.7	1	0	5	< 0.001
	2	145	2.5	1.6	2	0	5	
	3	145	2.9	1.5	3	0	5	
	4	145	3.0	1.6	3	0	5	
Q6. Over the past 3 days, have you felt at peace?	1	145	1.8	1.5	2	0	5	< 0.001
	2	145	2.4	1.5	2	0	5	
	3	145	2.7	1.5	3	0	5	
	4	145	2.8	1.5	3	0	5	
Q7. Have you had enough help and advice for your family to plan for the future?	1	144	2.9	1.9	3	0	5	< 0.001
	2	144	3.7	1.3	4	0	5	
	3	142	4.0	1.2	4	0	5	
	4	143	4.1	1.2	5	0	5	
Q8. How much information have you and your family been given?	1	142	3.3	1.6	3	0	5	< 0.001
	2	141	4.0	1.1	4	1	5	
	3	142	4.4	0.8	5	1	5	
	4	143	4.6	0.7	5	2	5	
Q9. How confident does the family feel caring for ____?	1	141	4.1	1.2	5	0	5	< 0.001
	2	140	4.3	1.0	5	1	5	
	3	141	4.5	0.9	5	1	5	
	4	142	4.5	0.9	5	1	5	
Q10. Has the family been feeling worried about the patient over the last 3 days?	1	142	3.1	1.6	3	0	5	< 0.001
	2	141	2.6	1.5	3	0	5	
	3	141	2.2	1.4	2	0	5	
	4	143	2.1	1.6	2	0	5	

Multiple themes and subthemes arose from the nurse interviews, including the challenge of pain management without access to morphine; the psychological burden on the caregivers; advocacy for oral morphine; the value of holistic care for patients and family; and improved pain management for most patients. The nurses all provided examples of observing patients’ pain being eased and emotional and spiritual well-being improving, even if pain was not completely relieved over time. In the interests of complete confidentiality, no demographic data were recorded for the nurses, who were given study numbers for reference purposes.

At a funeral for a patient attended by the palliative care team, families shared their appreciation:

‘They felt that the medications for pain made a real difference. They did not see that the pain was as bad as when she had been hospitalised, even when her condition was worsening.’ (Nurse 2)

Nurse 1 added:

‘Many are so happy to be visited and listened to, because we really try hard to address all of their problems, and comfort them even when they are in pain.’

Although pain reduction is a primary service, the emotional and psychological support provided to patients and their families can be seen in these quotes as well as the POS scores.

The value of the holistic nature of a palliative care approach in a context of limited resources was best summarised by this quote:

‘With our African setup you cannot simply say that now I managed the physical pain and everything is OK. There are other aspects of pain like the social pain, the spiritual pain and so on. They contribute a lot to pain and so if you are able to manage these other pains like the social pain and the spiritual pain and psychosocial pain then you will find that to some extent a lot of these pains are associated with the physical. For example, there might be family conflicts, or poverty, the child is not going to school, the breadwinner is unable to work; so you find some of these elements are adding pain into the physical pain.’ (Nurse 3)

All six nurses however, felt that their pain management would be improved by having access to oral morphine. Four of the six nurses trained to use morphine for severe, chronic pain expressed concern over not having access to potent pain relievers. Each nurse could easily tell the story of one or more patients who had suffered considerable unrelieved pain before death. Table 4 provides quotes from the nurses which exemplify some of these challenges of patient care and the psychological burden on the nurses.

**TABLE 4:** Nurse interview quotes.

Theme	Quote
**Caregiver stress**	‘It is a challenge [*not having morphine*], actually a very big one. You get there and you have nothing with which to help the pain. Sometimes the person needs help, and when you fail to deliver that help, it is as if you have done nothing. They may even wonder why you bothered to visit.’ (Nurse 5)
	‘As a PC [*palliative care*] professional you can only be satisfied when you feel you are doing the right thing, although you are not curing you are doing the right thing. But if you are not doing the right thing [*giving oral morphine*] you feel as if you are mismanaging the patient.’ (Nurse 3)
**Unrelieved pain**	‘For this case, it was very difficult to control her pain using tramadol and ibuprofen. There was not much improvement. It became necessary for me to go to KCMC [*regional hospital*] where we were able to get oral morphine. She was so happy to get that medication! She continued with it … but near the end she was unable to get it again, as we were unable to refill the medication. So we used tramadol and others as we had done before. It did not give her relief like when she had the morphine. The challenge was, if only in our institutions morphine would be available, in order that for patients like her with strong pain they might get some relief…Truly, I felt very badly; terrible. I kept thinking that there was medicine just there at KCMC. But the restrictions of the pharmacy, that they cannot just give it to anybody, it really hurt me. If only this woman had lived somewhere like Arusha, being near a place like Selian, where oral morphine is available without severe restriction.’ (Nurse 6)
	‘... a patient who was 70 years old or so, who had been found to have cancer of the prostate ... We worked with him with the pain. We started Diclopar [*diclofenac plus paracetamol*] four times daily and tramadol 50 mg four times daily. It did not help much ... Still the pain continued ... We had to increase pain medications, the tramadol to 100 mg four times daily. But 3 days later we found the pain to still be so much. More medications were added... amitriptyline and diazepam at night. From then pain reduced slightly but not a great deal. He continued until he was called by God... These patients are here. They do not respond satisfactorily to our pain medications and are not satisfied with our care.’ (Nurse 5)
	37 y.o. female with advanced ovarian cancer taking tramadol orally. ‘She reported to us with very advanced disease and very advanced pain; it was too late to really help her ... But all along her pain was not responding ... When she recognised what was going on, she didn't want to see us ... We had to gradually convince them that our services would help; it took time. Then when we did the pain did not improve. It began as overwhelming and was helped only slightly by our medications. Eventually she was unable to swallow and she died... That last time when she could not swallow tramadol, perhaps morphine in the mouth might have been absorbed and she could have had the chance of relief.’ (Nurse 4)

The nurses’ collective frustration with the limited access to oral morphine also proved to be an incentive for advocacy. Each of them spoke of lobbying within their own hospital with doctors and pharmacists. In addition, they spoke of the need to reduce government bureaucracy so that more hospitals could provide morphine. Nurse 5 spoke passionately:

‘My thoughts and advice is that the relevant ministries and government leaders should make the process of getting pain medications to be fair and easy. After all it is a basic human right for patients to get that service.’ (Nurse 5)

## Discussion

Our results indicate that even in the absence of morphine there is a benefit in providing palliative care to cancer patients. Overall pain scores improved from an average of 3.8 out of 5 (moderately severe pain) to 2.3 out of 5 (moderate pain) and non-pain symptoms and other indicators – psychological, spiritual and family issues – improved significantly as well. Both women and men experienced similar decreases in pain and positive changes in the other domains over the period of the four visits. However, the average last visit POS score still indicates a moderate degree of pain and is higher than that reported by facilities using morphine.^26, 27^ For example, in South Africa average APCA African POS scores went from 4 to 1 over six visits with morphine compared with our results of 4 to 2 with no morphine.^27^ This suggests the value of holistic palliative care to patients without access to morphine. However, without a breakdown by disease process it is difficult to assess how comparable the two populations are. The nurses, despite their frustration at not having access to oral morphine, nonetheless affirm the value of palliative care in the absence of morphine. In this rural Tanzanian setting palliative care is valuable and offers a significant degree of pain control in the absence of oral morphine, but not to the degree that could be achieved if morphine was available. Given that much of the world currently lacks access to a strong opioid for treating cancer pain,^28^ this finding has significance. Recent availability of the APCA African POS as a validated tool makes such assessment feasible. There are few examples of results in the peer-reviewed literature using the APCA African POS.^26, 27^ This study contributes to a nascent body of literature on this tool in different African settings.

From the nurse interviews there is, as a result of their training, a clear belief that pain control would be even better with oral morphine. All recalled difficult pain cases which might have been better managed had they only been able to access morphine. There is a burden to the team in having knowledge of optimal treatment without being able to deliver it. The psychological hardship for palliative and other community teams in resource-constrained environments is an area that has been under-researched.

There are a number of limitations to this study. We did not have access to a comparison hospital site with access to morphine and using POS – such direct comparison would have been very useful. Having only one investigator doing interviews and translations raises the possibility of bias and error. For the language of the POS, whilst Swahili is the dominant language in Tanzania there are more than 120 distinct languages in the country, some of which are in use amongst the 13 rural sites of this study. Translations from Swahili into the local language were occasionally done on the spot by the health professional administering the POS, or through a family interpreter.

Nonetheless, our results are in line with data from other palliative care providers in Africa. Loy et al.'s work^29^ shows the positive impact of palliative care for HIV clients and the nurse interviews in this study point out that clients have a great appreciation and sense of improvement when receiving palliative care. In South Africa, with access to oral morphine and where the POS is used regularly, average pain scores improved from 4 to 1 over time.^27^ Detailed data comparison from South Africa and APCA would be an area for further study, with the hypothesis that pain control is improved significantly with the availability of oral morphine.

Pursuit of oral morphine is an ongoing challenge for many palliative care advocates around the world. The mention of advocacy by several of the nurses suggests that there appears to be little danger of palliative care teams abandoning their pursuit of oral morphine access. This study, and indeed the CHAT project with its associated emphasis on quality measurement, is an important advocacy tool for accessing oral morphine and for palliative care in general. The availability of client-centred data is valuable in care provision, particularly in areas such as rural Tanzania where information is limited. In this regard the POS is an invaluable tool.^30, 31, 32^

The information in this and similar studies can be used to lobby government decisions regarding health policy. As Mmbando described in the APCA newsletter of April 2011,^33^ and as was written up in the FHSSA newsletter,^34^ it is as a result of this very emphasis on quality and advocacy that 12 out of 13 CHAT sites (including all sites from which the nurse interviewees came) had, as of February 2011, been granted approval for oral morphine usage by the Tanzania Food and Drug Authority. A year later (January 2012), continued advocacy and training brought morphine access to a total of 19 ELCT facilities, including all of the CHAT sites. With other programmes such as Muheza in Tanga Region and Tearfund in the Lake Zone^35^ having gained access to oral morphine, the prospects for pain management in rural Tanzania are much improved. By the end of 2012, there were 50 facilities – a mix of public and private – which had gained approval for oral morphine use through the Tanzania Food and Drug Authority (Paul Mmbando, personal communication, August 16, 2013). Although no funding is available at this time, future research in these same hospitals with similar patient populations to this study would be desirable in order to observe if there are differences in POS results now that morphine is more widely available.

## Conclusion

This study shows that palliative care without morphine is beneficial to cancer patients, but comparison with other studies suggests that pain control would be even better with access to oral morphine. A degree of pain relief for most cancer patients can be accomplished without strong opioids in the context of palliative care. A trained palliative care team, equipped with education and medications, can be a powerful advocate for people at the end of life, whether the disease be cancer, HIV, or any other. Effectiveness of the care, whether for pain or other domains of palliative care, can be measured and shown to be of value for clients and their families, but there is a psychological burden for nurses trained in palliative care and the use of morphine when they are asked to deliver palliative care without access to the world's most available strong analgesic.

Whilst palliative care team leaders can become excellent advocates for accessing oral morphine, more research is warranted in the area of supporting such teams that often operate without the full range of tools necessary to meet all of their patients’ needs.
